# Paralysis efficiency (PD_50_) scales linearly with lethality (LD_50_) in spider venoms

**DOI:** 10.1016/j.toxcx.2026.100256

**Published:** 2026-04-17

**Authors:** Keith Lyons, Dayle Leonard, Leona McSharry, Michael Martindale, Brandon L. Collier, Aiste Vitkauskaite, John P. Dunbar, Michel M. Dugon, Kevin Healy

**Affiliations:** a Macroecology Lab, School of Natural Sciences, Ryan Institute, University of Galway, H91 TK33, Galway, Ireland; b Venom Systems & Proteomics Lab, School of Natural Sciences, Ryan Institute, University of Galway, H91 TK33, Galway, Ireland; c Institute for Insect Biotechnology, Faculty for Agricultural Sciences, Nutritional Sciences, and Environmental Management, Justus Liebig University Giessen, Heinrich-Buff-Ring 58, Giessen, 35392, Germany; d Midlands Bug and Reptile Zoo, Longford, Ireland

**Keywords:** Spider, Venom potency, LD_50_, PD_50_, Bioassays, Potency measures, Venom yield

## Abstract

Historically, venom potencies have been assessed using measures of lethality, such as the median lethal dose (LD_50_). However, venoms may be selected primarily for their ability to rapidly incapacitate rather than cause mortality, meaning LD_50_ may not capture the efficacy of venoms in an ecological and evolutionary context. To capture this context, recent studies have adapted measures that assess venoms’ ability to rapidly incapacitate, such as the median paralysis dose (PD_50_). However, while PD_50_ values are expected to provide a more proximate assessment of ecological variation in venom potency, it is unknown whether historically available LD_50_ values are still useful proxies of ecologically relevant potency or whether they capture independent axes of venom variation. Here, we test the relationship between LD_50_ and PD_50_ in spider venoms by experimentally estimating LD_50_ and PD_50_ for 12 species and collating additional potency data for 46 species retrieved from the literature, producing a dataset of 55 species spanning 26 families when combined. We observed a linear isometric relationship between LD_50_ and PD_50_, showing these potency measures are both strongly correlated, with an increase in paralysis efficiency associated with a similar increase in lethality. Our results suggest that due to the correlation between functional aspects of venom potency, paralysis and lethality, historically available LD_50_ values may be used to compare general venom potencies in spiders, provided that they are based on the same prey model.

## Highlights

• We found an isometric relationship between PD_50_ & LD_50_ in 55 spider species venoms.
• The main analysis included data produced in the lab & compiled literature data.
• Spider venom PD_50_ & LD_50_ values may be used as proxies for one another.

## Graphical abstract

**Figure:**
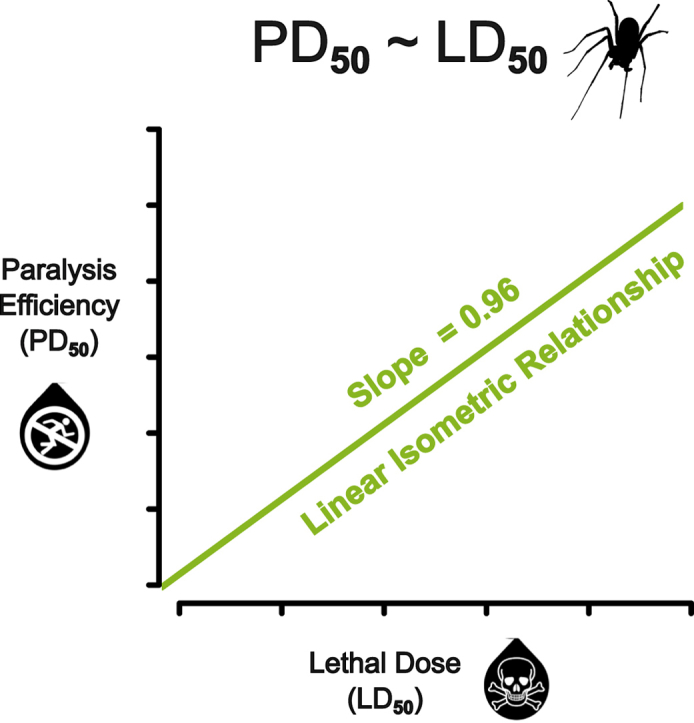

## 1 Introduction

Venom has evolved independently across the Animal Kingdom more than 100 times, in at least eight phyla and is involved in at least 14 distinct ecological roles (Schendel et al., 2019; Hayes et al., 2025). Predation is one of the more ubiquitous roles, functioning as the primary role of venoms in cephalopods, centipedes, cnidarians, cone snails, heteropterans, scorpions, snakes and spiders, among others (Arbuckle, 2017; Schendel et al., 2019; Hayes et al., 2025; Herzig, 2025; Jenner et al., 2025). Animals with predation-oriented venoms have evolved toxins that function both individually and synergistically to manipulate important physiological and signalling processes in their prey, resulting in non-lethal, detrimental effects or death while facilitating consumption (Arbuckle, 2017; Kuhn-Nentwig et al., 2019; Schendel et al., 2019; Jenner et al., 2025). These toxins employ various mechanisms to subdue prey, including haemotoxins that enhance or disrupt blood clotting factors and lyse erythrocytes, necrotoxins (i.e., cytotoxins) that cause focal to locally extensive tissue necrosis, and neurotoxins that target the nervous system and induce both rapid and prolonged paralytic effects (Kuhn-Nentwig et al., 2019; Bolon et al., 2023). While these toxins can cause prey mortalities (Herrera et al., 2012; Brinkman et al., 2014), it is hypothesised that predation-oriented toxins are selected primarily for their ability to rapidly incapacitate prey (Arbuckle, 2017; Michálek et al., 2024). This is because achieving prey mortality in an ecologically relevant timeframe may require more venom than is required to incapacitate, incurring higher metabolic costs, whereas rapid paralysis is often sufficient to facilitate mechanical dispatch and the consumption of prey (Wigger et al., 2002; Morgenstern and King, 2013; Arbuckle, 2017). However, classical potency measures have focused primarily on lethality, potentially missing ecologically relevant patterns associated with venom potency.

To better understand how venom potency has evolved, researchers use various measures which typically involve introducing a known amount of venom or specific venom fractions, serially diluted to multiple concentrations, into a prey model and recording the resulting effects (Finney, 1985). Historically, the most common measure of venom potency is the median lethal dose (Finney, 1985) or LD_50_, the amount of venom required to cause a 50% mortality rate in a prey model cohort (Bücherl, 1946; Lyons et al., 2025). Although many venoms can cause mortality in prey models, it typically takes considerably longer than the initial paralysis, suggesting the mortality rates recorded during LD_50_ assays may not be ecologically relevant (Michálek et al., 2024). Consequently, recent studies have adapted alternative measures such as the median paralysis dose (Pearce et al., 1995) or PD_50_ (McElroy et al., 2017; Michálek et al., 2022; Lyons et al., 2023) which measures the amount of venom required to incapacitate 50% of a prey model cohort. While LD_50_ typically gauges the capacity of venoms to cause prey mortality within 24 h (Michálek et al., 2024), PD_50_ usually gauges their ability to incapacitate prey within more ecologically relevant timeframes, which vary between studies—30 min (Rayner et al., 2022), 1 h (Michálek et al., 2019), 4 h (Lyons et al., 2023), 5 h (Manzoli-Palma et al., 2003) and rarely more than 24 h (Herzig and Hodgson, 2008). Hence, PD_50_ is expected to be better suited for detecting novel, ecologically relevant aspects of venom potency. However, while PD_50_ values may capture more ecologically relevant variation, they are not as taxonomically widespread in the literature or as frequent as lethality measures, such as LD_50_, raising the question of whether such historically common measures should be used in comparative studies addressing ecological and evolutionary questions. The use of such historical lethality measures to better understand ecological patterns in venom potency may thus be dependent on whether the ability of venoms to incapacitate prey quickly is generally decoupled from subsequent lethality, or coupled, with selection for an increase in paralysis efficiency resulting in a similar increase in lethality.

In the context of evolutionary biology, functional coupling is typically associated with a physiological trait or set of traits that perform multiple, overlapping functions (Farina et al., 2019). For example, the elytra of beetles are modified forewings used in defence, flight and a plethora of other functions (Linz et al., 2016; Goczał and Beutel, 2023), while the cranial components of teleost fish are functionally coordinated to perform the overlapping functions of suction feeding and gill ventilation (Farina et al., 2019). However, not all traits or functions in functional coupling are directly selected for. For example, species that change their body sizes due to selection are likely to display changes in the top speed at which they can move, not necessarily due to direct selection for speed but more likely due to the physiological constraints linked with size (Mezzini et al., 2025). Likewise, different functional aspects of venom potency, such as those measured by PD_50_ and LD_50_, may be tightly linked due to strong mechanistic coupling. For example, neurotoxins selected for their ability to rapidly incapacitate prey (Escoubas et al., 2000; Osipov and Utkin, 2015; Gao et al., 2017) may demonstrate strong links with mortality as paralytic effects often involve the disruption of vital functions which can ultimately lead to organ failure (Langenegger et al., 2019; Bolon et al., 2023). However, such immediate effects of venoms may not necessarily be linked to the later onset of mortality, with many venom fractions inducing rapid but reversible paralysis in prey (Loret et al., 1992; Adams, 2004; Michálek et al., 2022). Hence, determining whether there is a relationship between PD_50_ and LD_50_ values, thereby implying a link between the functions they measure, would not only help ascertain the potential value of historic LD_50_ values for inferring ecological patterns in venoms but would also improve our understanding of the general mechanisms underlying the functionality of predation-oriented venoms.

Through the combination of experimental data produced in the lab for 12 species and data collated from the literature for 46 species, we test the relationship between LD_50_ and PD_50_ for 55 spider species spanning 26 families via a phylogenetic comparative analysis. While we expect PD_50_ values to be far lower than their corresponding LD_50_ values, as less venom is typically required to achieve prey paralysis compared to prey mortality (Wigger et al., 2002; Morgenstern and King, 2013), we predict that if LD_50_ and PD_50_ are functionally coupled, they will scale isometrically with a slope close to one. Alternatively, if LD_50_ and PD_50_ are functionally decoupled, they will instead have a sublinear relationship, with expected LD_50_ efficiencies decreasing faster than their respective PD_50_ efficiencies in the scenario where there are higher selective pressures for prey paralysis compared to lethality.

## 2 Materials and methods

### 2.1 Animals and husbandry

We collected six of the twelve spider species studied, *Amaurobius similis* (Lace-Weaver), *Eratigena atrica* (Giant House Spider), *Larinioides sclopetarius* (Bridge Spider), *Meta menardi* (European Cave Spider), *Pholcus phalangioides* (Cellar Spider) and *Steatoda nobilis* (Noble False Widow) between August and November of 2021 and 2022, in sites between Co. Galway and Co. Sligo in Ireland. The other six species: *Heteropoda venatoria* (Giant Crab Spider), *Monocentropus balfouri* (Socotra Island Blue Baboon Tarantula), *Phormictopus cancerides* (Hispaniola Giant Tarantula), *Piloctenus haematostoma* (Guinean Wandering Spider), *Cupiennius coccineus* and *Cupiennius salei* (Bromeliad spiders) were either donated by Mark Stockmann, a captive breeder and owner of Buthidae.eu based in Hörstel, Germany or purchased from “The Pet Factory”, also based in Germany. All spiders were kept at constant room temperature (21 °C), with the exception of *M. balfouri*, *P. cancerides*, *H. venatoria*, *P. haematostoma* (27 °C) and *M. menardi,* which was stored at 10 °C with moderate humidity (60-70%) as they are prone to desiccation (Howarth, 1983). All spiders were not fed for two weeks before venom extraction to allow for adequate venom production, as per Boevé et al. (1995).

House crickets (*Acheta domesticus)* were purchased from retailers in Co. Galway while Common rough woodlice (*Porcellio scaber*) were collected from stone walls in Co. Galway. Specimens were selected based on their weight to fall within the range of 0.1 g – 0.5 g (median weight of 0.3 g per cohort) for *A. domesticus* and within the range of 0.06 g – 0.12 g (median weight of 0.09 g per cohort) for *P. scaber*. Prior to the bioassays, crickets and woodlice were housed in separate, large plastic boxes with egg cardboard hides (crickets) or bark covered with moss (woodlice) and provided with moistened pieces of peeled organic carrot for a minimum of 24 h at 21 °C.

### 2.2 Spider venom extractions

Venom was extracted from all spiders through electrical stimulation as described in Lyons et al. (2023), except for *P. phalangioides,* for which venom was obtained by excising the venom glands (Lyons et al., 2023). For all species, the venoms of both males and females were pooled. Venom was extracted from each spider only once to minimise effects of multiple extractions on the venom samples except for both *Cupiennius* spp. and *H. venatoria*, for which a second extraction was performed one month after the original extraction. Venom samples were flash frozen in liquid nitrogen, freeze-dried in a lyophiliser and stored at −80 °C until required for bioassays (S1, S5: Table S1).

### 2.3 Spider venom bioassays

To determine the median lethal dose (LD_50_) (Finney, 1985) and median paralysis dose (PD_50_) (Pearce et al., 1995) for each species venom, *in-vivo* bioassays were conducted in cricket and woodlice cohorts of 20 individuals each (n = 20). First, lyophilised venom samples from each spider species were weighed and rehydrated with 0.01 M Phosphate-buffered saline (PBS) to the original concentration of the crude venom initially extracted (See S1: column AN & AO for original concentrations in μl and mg). Venom concentrations for each species were then determined based on the amount of venom available, starting as high as possible while allowing for serial dilution of the venom to at least two lower concentrations (S1, S5: Table S6). For each venom concentration and prey model cohort, 5 μL (μl) of venom per specimen was injected using a 50 μl microsyringe (Hamilton Neurosyringe, model 1705RN, point style 3). Crickets were injected intrathoracically between the middle and hind leg, 2 mm (mm) deep while woodlice were injected ventrally, two mm deep, just posterior of the genital papilla on the first pleopod. Once injected, crickets and woodlice were maintained individually in petri dishes with damp paper towel and were checked for signs of paralysis or death at set intervals (1, 3, 5, 7, 10, 15, 30, 60, 120, 240 and 1440 min). Paralysis was recorded when a specimen was unable to move normally after a light touch with forceps (Michálek et al., 2019) and could not right itself within 1 min after being flipped upside down (Khamtorn et al., 2020). Cricket and woodlice mortality was recorded if no response to gentle stimulus was observed from the moment the test was initiated until the end of the experiment (24 h). After the initial three concentrations were tested in both prey models, for each species venom, additional venom concentrations were tested until there was insufficient venom to produce another viable concentration or the previous venom concentration tested proved ineffective (S1, S5: Table S6). Two control cohorts (n = 20) for each prey model were also tested using 0.01 M PBS instead of venom, with all crickets and woodlice surviving 24 h post-injection and no signs of paralysis noted throughout the experiments.

### 2.4 *LD*_*50*_*and* PD_50_*Calculations*

To calculate each LD_50_ and PD_50_ value in milligrams of venom per kilogram of prey mass (mg/kg), we used Probit models from the Mass package (Venables and Ripley, 2013) in R version 4.5.0 (Team, 2022) (S1, S3). The LD_50_ value for each species venom, per prey model, was calculated using the number of deaths that occurred within 24 h, per venom concentration tested, as per Lyons et al. (2023). The PD_50_ value for each species venom, per prey model, was calculated using the maximum number of paralysed individuals reached for each venom concentration within 4 h, as 4 h is one of the later times reported for the effects of the paralysis to conclude and for lethal effects to begin (Herzig and Hodgson, 2008). In addition, supplementary PD_50_ values were calculated using the maximum number of paralysed individuals reached for each venom concentration within 1 h, the most frequently used timepoint in the literature (Quistad et al., 1992; Dutertre et al., 2014; Michálek et al. 2019, 2022), to determine if the difference between 1 h and 4 h as endpoints notably changes the resulting PD_50_ values and the relationship between LD_50_ and PD_50_ (S1, S3, S5: Table S2).

### 2.5 Data and phylogeny for phylogenetic comparative analysis

We collated additional LD_50_ and PD_50_ values from the literature and combined the data with the values produced in the lab for 12 species to perform one large scale phylogenetic comparative analysis. Only potency values measured in microliters of venom per gram of prey mass (μl/g), microgram of venom per gram of prey mass (μg/g) or values convertible to these units were included as they were the most prevalent units of measurement for spider potency values in the literature. Furthermore, potency values were only included if a PD_50_ value had a corresponding LD_50_ value for the same species venom and both values were tested in the same prey model species (S2).

To build a phylogeny to account for non-independence of data due to common ancestry in our phylogenetic comparative analysis, we used Wolff et al. (2022) as a backbone phylogeny, following the approach used in Lyons et al. (2025). For species not present in Wolff's phylogeny, we inferred their position based on phylogenetic evidence from the literature (Maddison and Hedin, 2003; Edwards, 2004; Bolzern et al., 2013; Garrison et al., 2016; Azevedo et al., 2018; Ortiz et al., 2018; Piacentini and Ramírez, 2019; Briggs et al., 2023; Guerrero-Fuentes et al., 2024) (S4). All steps performed to produce the final phylogeny were completed in R version 4.5.0 (Team, 2022) (S2, S4, S5: Fig. S5).

### 2.6 Statistical analyses

To determine the relationship between LD_50_ and PD_50_ in 55 spider species, including data produced in the lab and data collated from the literature, we fitted a Bayesian phylogenetic mixed model, using the Markov chain Monte Carlo generalized linear mixed model (MCMCglmm) package (Hadfield, 2010) in R version 4.5.0 (Team, 2022), which allows for the inclusion of variance terms to account for multiple observations per species and the inclusion of a phylogenetic term (Hadfield, 2010). We controlled for pseudoreplication due to shared ancestry between species through the ‘animal’ term, which uses a distance matrix of the phylogenetic distance between species to control for the expected similarity in factor values. We then calculated the relative variance attributable to the animal term as h^2^, which can be interpreted similarly to the phylogenetic lambda value (Hadfield and Nakagawa, 2010). To include multiple LD_50_ and PD_50_ measures for each species in our analysis, we used a random term for species, similar to previous comparative models of venom variation (Healy et al., 2019; Lyons et al. 2020, 2025). As the MCMCglmm is a Bayesian approach that requires specifying priors, we fit all models using standard, flat, non-informative priors, which assume no prior expectations for the estimated values across the model. We used a burn-in of 40 000 and a thinning of 100 over 2 400 000 iterations to ensure that the sample sizes exceeded 1000 for all parameter estimates. We tested for convergence using the Gelman–Rubin statistic over three separate chains (Hadfield, 2010). Significance of an estimate is determined when the 95% credibility interval (CI) does not cross zero (Hadfield, 2010). A supplementary MCMCglmm (model S4) was run using the data collated from the literature only in order to determine if a similar relationship to the main analysis would be observed if the experimental data produced in the lab was excluded (S5: Table S4). In both the main model and model S4, we included log_10_ of PD_50_ as the response variable and log_10_ of LD_50_ as the independent variable (S5).

Three supplementary Generalized linear models (GLM) (Dey et al., 2000) were fitted using the ‘stats’ statistical package in R version 4.5.0 (Team, 2022) (S1, S3). We included the prey model used to measure potency (*A. domesticus*, *P. scaber*) as a categorical factor in each GLM. The first supplementary GLM determined if a similar relationship to the main analysis would be observed when testing the relationship between PD_50_ (4 h endpoint) and LD_50_ in the experimental (12 species) data only (S2, S3, S5: Table S1). The second supplementary GLM tested the relationship between LD_50_ and PD_50_ using PD_50_ values calculated with the 1 h endpoint in order to determine if using a different endpoint changed the scaling relationship between LD_50_ and PD_50_ (S1, S3, S5: Table S2). The third supplementary GLM tested the relationship between LD_50_ and PD_50_ for 11 species, with *P. phalangioides* excluded as its venom was extracted using a different method, which can potentially affect a spider's PD_50_ value (Lyons et al., 2023) (S1, S3, S5: Table S3). In all of the models, we included log_10_ of PD_50_ as the response variable and log_10_ of LD_50_ as the independent variable (S5).

## 3 Results

### 3.1 Data description and tables

In the lab, the potency of 12 spider species venoms from nine different families (Amaurobiidae, Trechaleidae, Agelenidae, Sparassidae, Araneidae, Tetragnathidae, Theraphosidae, Pholcidae and Ctenidae) was gauged, with a total of 48 venom potency measures (24 LD_50_ mg/kg (24 h) and 24 PD_50_ mg/kg (4 h)) performed on two prey models, crickets (*A. domesticus*) and woodlice (*P. scaber*) (Table 1, S1). LD_50_ values in crickets ranged from 0.72 mg/kg for *S. nobilis* venom to 58.85 mg/kg for *P. cancerides* venom while LD_50_ values in woodlice ranged from 7.47 mg/kg for *S. nobilis* venom to 300.54 mg/kg for *E. atrica* venom. PD_50_ (4 h) values in crickets ranged from 0.036 mg/kg for *S. nobilis* venom to 16.7 mg/kg for *P. cancerides* venom while PD_50_ (4 h) values in woodlice ranged from 0.87 for *S. nobilis* venom to 28.06 mg/kg for *P. cancerides* venom (Table 1, S1).

**Table 1:** **Calculated potency values for 12 spider species venoms** tested on house cricket *A. domesticus* and woodlouse *P. scaber*. Presented in bold here are the PD_50_ (mg/kg) (4 h endpoint) and LD_50_ (mg/kg) (24 h endpoint) values for each species venom, in both prey models. Standard errors were calculated using the lyophilised (dried) venom protein mass (mg) for each species venom sample and the median prey model mass for crickets (0.0003 kg) or woodlice (0.00009 kg). *A. domesticus* data for *Eratigena atrica* venom was produced as part of Lyons et al. (2023). Each species venom yield, expressed in milligram of dried venom extracted per specimen (mg/spider), is also presented. For PD_50_ (1 h endpoint) values, see supplementary document S5: Table S7.

Spider Species	Venom Yield (mg/spider)	*Acheta domesticus* (cricket)				*Porcellio scaber* (woodlouse)			
		PD_50_ mg/kg	Standard Error	LD_50_ mg/kg	Standard Error	PD_50_ mg/kg	Standard Error	LD_50_ mg/kg	Standard Error
*Amaurobius similis*	0.0094	**1.9**	0.61	**9.0**	2.35	**10.2**	6.86	**33.5**	17.67
*Cupiennius coccineus*	0.04	**4.8**	1.24	**10.5**	6.71	**25.7**	20.19	**26.4**	10.86
*Cupiennius salei*	0.038	**2.4**	0.66	**6.4**	1.31	**25.1**	12.59	**37.2**	19.93
*Eratigena atrica*	0.065	**5.6**	2.52	**54.6**	10.66	**22.8**	17.13	**300.5**	50.23
*Heteropoda venatoria*	0.33	**1.0**	0.14	**37.6**	6.68	**17.0**	8.14	**131.5**	57.16
*Larinioides sclopetarius*	0.0205	**2.8**	0.46	**15.1**	6.87	**26.2**	3.34	**37.3**	15.18
*Meta menardi*	0.019	**0.7**	0.20	**12.5**	2.74	**3.2**	2.88	**75.0**	58.5
*Monocentropus balfouri*	0.55	**3.0**	0.92	**15.2**	1.66	**8.1**	6.39	**8.5**	6.51
*Pholcus phalangioides*	0.0046	**0.8**	0.41	**6.3**	2.25	**4.5**	1.64	**12.6**	1.56
*Phormictopus cancerides*	1.83	**16.7**	3.56	**58.8**	53.94	**28.1**	3.87	**100.3**	98.5
*Piloctenus haematostoma*	0.075	**2.7**	0.57	**5.9**	1.88	**10.3**	3.14	**19.2**	9.47
*Steatoda nobilis*	0.012	**0.04**	0.033	**0.7**	0.20	**0.9**	0.82	**7.5**	6.7

The data collated from the literature included 46 spider species spanning 23 different families, with a total of 130 venom potency values in μl/g and μg/g (65 LD_50_ and 65 PD_50_), with varying endpoints (30 min to 72 h) used to calculate the PD_50_. However, most PD_50_ values in the literature based dataset were calculated using the 1 h endpoint (S2). LD_50_ values measured in μl/g ranged from 0.003 μl/g for *Haplodrassus sp.* venom tested on Tobacco hornworm *Manduca sexta* to 8.52 μl/g for *Stegodyphus lineatus* venom tested on *Pardosa* sp. spiders. PD_50_ values measured in μl/g ranged from 0.00017 μl/g for *Latrodectus hesperus* venom tested on Oriental cockroach *Blatta orientalis* to 10.4 μl/g for *Scytodes sp.* venom tested on *M. sexta* (Table 2, S2). LD_50_ values measured in μg/g ranged from 0.46 μg/g for *E. atrica* venom tested on the Noble false widow *S. nobilis* to 445.3 μg/g for *A. similis* venom tested on Giant house spider *E. atrica*. PD_50_ values measured in μg/g ranged from 0.71 μg/g for *E. atrica* venom tested on *S. nobilis* to 152.7 μg/g for *Selenotholus foelschei* venom tested on House cricket *A. domesticus*.

**Table 2:** **Collated PD_50_ and LD_50_ values from the literature, including 130 measures (65 PD_50_ and 65 LD_50_) for 46 species spanning 23 families are provided in bold.** The data includes potency values measured in two different units (μl/g or μg/g) and 11 different prey models (eight insects, three arachnids). We also include reported measurements of uncertainty as either standard error (S.E.), 95% confidence levels (95% CL) or 95% confidence intervals (95% CI) depending on which measure was reported in the source. The source of the original data for each species is listed in the references column (for full citations see bibliography section). See dataset S2 for additional information.

Spider Species	PD_50_	Measurement of Uncertainty	LD_50_	Measurement of Uncertainty	Unit	Prey Model	Reference
*Agelenopsis aperta*	**3.1**	2.5 - 4.0 (95% CL)	**2.8**	2.3 - 3.4 (95% CL)	μl/g	*Manduca sexta*	Quistad et al. (1992)
*Agelenopsis aperta*	**4.2**	3.2 - 5.5 (95% CL)	**2.8**	2.0 - 4.0 (95% CL)	μl/g	*Diabrotica undecimpunctata undecimpunctata*	Quistad et al. (1992)
*Amaurobius similis*	**10.07**	0.26 (S.E.)	**445.3**	0.5 (S.E.)	μg/g	*Eratigena atrica*	Rayner et al. (2022)
*Aphonopelma chalcodes*	**0.59**	0.45 - 0.79 (95% CL)	**0.63**	0.42 - 0.94 (95% CL)	μl/g	*Manduca sexta*	Quistad et al. (1992)
*Atrax robustus*	**0.0043**	na	**0.137**	na	μl/g	*Blatta orientalis*	Nentwig et al., 1992
*Atrax robustus*	**0.0026**	na	**0.4**	nan	μl/g	*Blatta orientalis*	Nentwig et al., 1992
*Avicularia avicularia*	**0.001**	na	**0.1857**	na	μl/g	*Blatta orientalis*	Nentwig et al., 1992
*Calilena sp.*	**1.3**	0.96 - 1.7 (95% CL)	**0.64**	0.58 - 0.77 (95% CL)	μl/g	*Manduca sexta*	Quistad et al. (1992)
*Cheiracanthium mildei*	**0.08**	0.06 - 0.11 (95% CL)	**0.15**	0.1 - 0.22 (95% CL)	μl/g	*Manduca sexta*	Quistad et al. (1992)
*Cupiennius salei*	**0.014**	na	**0.2**	na	μl/g	*Blatta orientalis*	Friedel and Nentwig (1989)
*Cupiennius salei*	**0.397**	na	**1.842**	na	μl/g	*Tenebrio monitor*	Friedel and Nentwig (1989)
*Cybaeodamus taim*	**0.17**	0.07 - 0.4 (95% CI)	**1.82**	1.11 - 2.96 (95% CI)	μl/g	*Lasius flavus*	Michálek et al. (2019)
*Cybaeodamus taim*	**0.25**	0.15 - 0.44 (95% CI)	**0.96**	0.57 - 1.62 (95% CI)	μl/g	*Acheta domesticus*	Michálek et al. (2019)
*Diguetia albolineata*	**0.23**	0.16 - 0.32 (95% CL)	**0.33**	0.21 - 0.51 (95% CL)	μl/g	*Manduca sexta*	Quistad et al. (1992)
*Eratigena atrica*	**0.0017**	na	**0.057**	na	μl/g	*Blatta orientalis*	Nentwig et al. (1992)
*Eratigena atrica*	**0.71**	0.17 (S.E.)	**0.46**	0.23 (S.E.)	μg/g	*Steatoda nobilis*	Rayner et al. (2022)
*Filistata sp.*	**0.06**	0.04 - 0.1 (95% CL)	**0.008**	0.004 - 0.02 (95% CL)	μl/g	*Manduca sexta*	Quistad et al. (1992)
*Filistata sp.*	**0.7**	0.57 - 0.81 (95% CL)	**0.38**	0.29 - 0.49 (95% CL)	μl/g	*Diabrotica undecimpunctata undecimpunctata*	Quistad et al. (1992)
*Gnaphosa sp.*	**0.05**	0.03 - 0.07 (95% CI)	**0.28**	0.19 - 0.41 (95% CI)	μl/g	*Pardosa sp.*	Michálek et al. (2022)
*Gnaphosa sp.*	**0.05**	0.03 - 0.08 (95% CI)	**0.26**	0.14 - 0.48 (95% CI)	μl/g	*Gryllus assimilis*	Michálek et al. (2022)
*Hadronyche cerberea*	**8.5**	0.1 (S.E.)	**32.3**	7.0 (S.E.)	μg/g	*Lucilia cuprina*	Cardoso et al. (2022)
*Hadronyche cerberea*	**7.5**	0.2 (S.E.)	**52.2**	2.4 (S.E.)	μg/g	*Lucilia cuprina*	Cardoso et al. (2022)
*Hadronyche formidabilis*	**4**	1.1 (S.E.)	**64.3**	5.8 (S.E.)	μg/g	*Lucilia cuprina*	Cardoso et al. (2022)
*Hadronyche formidabilis*	**11.4**	0.0 (S.E.)	**85**	6.0 (S.E.)	μg/g	*Lucilia cuprina*	Cardoso et al. (2022)
*Hadronyche infensa*	**10.2**	0.2 (S.E.)	**17.8**	2.5 (S.E.)	μg/g	*Lucilia cuprina*	Cardoso et al. (2022)
*Hadronyche infensa*	**4.6**	0.8 (S.E.)	**11.5**	1.4 (S.E.)	μg/g	*Lucilia cuprina*	Cardoso et al. (2022)
*Hapalopus limensis*	**1.3**	0.39 - 1.8 (95% CL)	**0.45**	0.32 - 0.65 (95% CL)	μl/g	*Manduca sexta*	Quistad et al. (1992)
*Haplodrassus sp.*	**0.007**	0.005 - 0.01 (95% CL)	**0.003**	0.002 - 0.006 (95% CL)	μl/g	*Manduca sexta*	Quistad et al. (1992)
*Herpyllus propinquous*	**0.04**	0.03 - 0.05 (95% CL)	**0.03**	0.02 - 0.04 (95% CL)	μl/g	*Manduca sexta*	Quistad et al. (1992)
*Hogna carolinensis*	**1.1**	0.7 - 1.6 (95% CL)	**1.8**	1.2 - 2.7 (95% CL)	μl/g	*Manduca sexta*	Quistad et al. (1992)
*Hololena curta*	**1.1**	0.79 - 1.6 (95% CL)	**0.78**	0.52 - 1.2 (95% CL)	μl/g	*Manduca sexta*	Quistad et al. (1992)
*Kukulkania sp.*	**0.00037**	na	**0.0057**	na	μl/g	*Blatta orientalis*	Nentwig et al., 1992
*Lampona sp.*	**0.06**	0.04 - 0.09 (95% CI)	**0.07**	0.05 - 0.1 (95% CI)	μl/g	*Pardosa sp.*	Michálek et al. (2022)
*Lampona sp.*	**0.85**	0.59 - 1.23 (95% CI)	**3.27**	2.02 - 5.31 (95% CI)	μl/g	*Gryllus assimilis*	Michálek et al. (2022)
*Latrodectus hesperus*	**0.009**	0.005 - 0.016 (95% CL)	**0.007**	0.005 - 0.011 (95% CL)	μl/g	*Manduca sexta*	Quistad et al. (1992)
*Latrodectus hesperus*	**0.00017**	na	**0.0086**	na	μl/g	*Blatta orientalis*	Nentwig et al., 1992
*Loxosceles deserta*	**0.05**	0.04 - 0.07 (95% CL)	**0.03**	0.02 - 0.4 (95% CL)	μl/g	*Manduca sexta*	Quistad et al. (1992)
*Loxosceles deserta*	**0.0013**	na	**0.071**	na	μl/g	*Blatta orientalis*	Nentwig et al., 1992
*Metepeira sp.*	**2.2**	1.6 - 3.1 (95% CL)	**1.9**	1.5 - 2.6 (95% CL)	μl/g	*Manduca sexta*	Quistad et al. (1992)
*Olios sp.*	**0.0091**	na	**0.114**	na	μl/g	*Blatta orientalis*	Nentwig et al., 1992
*Orodrassus sp.*	**0.03**	0.02 - 0.04 (95% CL)	**0.02**	0.01 - 0.03 (95% CL)	μl/g	*Manduca sexta*	Quistad et al. (1992)
*Palpimanus sp.*	**0.01**	0.0 - 0.02 (95% CI)	**0.07**	0.06 - 0.09 (95% CI)	μl/g	*Pardosa sp.*	Michálek et al. (2019)
*Palpimanus sp.*	**0.13**	0.01 - 0.17 (95% CI)	**3.6**	1.84 - 7.02 (95% CI)	μl/g	*Acheta domesticus*	Michálek et al. (2019)
*Peucetia viridans*	**0.8**	0.6 - 0.98 (95% CL)	**1.5**	1.0 - 2.3 (95% CL)	μl/g	*Manduca sexta*	Quistad et al. (1992)
*Peucetia viridans*	**1.7**	1.1 - 2.4 (95% CL)	**2**	1.4 - 2.9 (95% CL)	μl/g	*Diabrotica undecimpunctata undecimpunctata*	Quistad et al. (1992)
*Peucetia viridans*	**0.00069**	na	**0.43**	na	μl/g	*Blatta orientalis*	Nentwig et al., 1992
*Phidippus ardens*	**0.23**	0.06 - 0.84 (95% CL)	**0.28**	0.14 - 0.55 (95% CL)	μl/g	*Manduca sexta*	Quistad et al. (1992)
*Phidippus audax*	**0.18**	0.15 - 0.23 (95% CL)	**0.2**	0.16 - 0.25 (95% CL)	μl/g	*Manduca sexta*	Quistad et al. (1992)
*Phidippus johnsoni*	**0.14**	0.1 - 0.2 (95% CL)	**0.17**	0.12 - 0.25 (95% CL)	μl/g	*Manduca sexta*	Quistad et al. (1992)
*Phidippus johnsoni*	**0.0014**	na	**0.069**	na	μl/g	*Blatta orientalis*	Nentwig et al., 1992
*Phidippus octopunctatus*	**1.2**	0.18 - 7.5 (95% CL)	**0.46**	0.19 - 1.1 (95% CL)	μl/g	*Manduca sexta*	Quistad et al. (1992)
*Philodromid sp.*	**0.1**	0.08 - 0.13 (95% CL)	**0.29**	0.24 - 0.37 (95% CL)	μl/g	*Manduca sexta*	Quistad et al. (1992)
*Plectreurys tristis*	**0.0017**	na	**0.069**	na	μl/g	*Blatta orientalis*	Nentwig et al., 1992
*Schizocosa mccooki*	**1.2**	1.0 - 1.3 (95% CL)	**1.3**	1.1 - 1.5 (95% CL)	μl/g	*Manduca sexta*	Quistad et al. (1992)
*Scytodes sp.*	**10.4**	5.8 – 19.0 (95% CL)	**7.1**	4.8 – 11.0 (95% CL)	μl/g	*Manduca sexta*	Quistad et al. (1992)
*Selenotholus foelschei*	**152.7**	na	**241.9**	na	μg/g	*Acheta domesticus*	Herzig and Hodgson (2008)
*Steatoda grossa*	**3.3**	2.4 - 4.8 (95% CL)	**2.4**	1.6 - 3.7 (95% CL)	μl/g	*Manduca sexta*	Quistad et al. (1992)
*Stegodyphus lineatus*	**8.66**	4.82 - 15.54 (95% CI)	**8.52**	4.86 - 14.95 (95% CI)	μl/g	*Pardosa sp.*	Michálek et al. (2019)
*Stegodyphus lineatus*	**0.27**	0.14 - 0.53 (95% CI)	**1.74**	0.96 - 3.17 (95% CI)	μl/g	*Acheta domesticus*	Michálek et al. (2019)
*Tigrosa helluo*	**0.91**	0.65 - 1.3 (95% CL)	**0.68**	0.52 - 0.89 (95% CL)	μl/g	*Manduca sexta*	Quistad et al. (1992)
*Tliltocatl albopilosus*	**0.0017**	na	**0.42**	na	μl/g	*Blatta orientalis*	Friedel and Nentwig. 1989
*Tliltocatl albopilosus*	**0.029**	na	**0.344**	na	μl/g	*Tenebrio monitor*	Friedel and Nentwig. 1989
*Trochosa sp.*	**2.5**	1.6 - 3.8 (95% CL)	**1**	0.75 - 1.4 (95% CL)	μl/g	*Manduca sexta*	Quistad et al. (1992)
*Zodarion nitidum*	**0.07**	0.06 - 0.08 (95% CI)	**0.06**	0.05 - 0.06 (95% CI)	μl/g	*Lasius flavus*	Michálek et al. (2019)
*Zygiella x-notata*	**2.39**	0.31 (S.E.)	**69.8**	0.16 (S.E.)	μg/g	*Eratigena atrica*	Rayner et al. (2022)

### 3.2 Relationship between PD_50_ and LD_50_ in spider venoms

**Fig. 1:**
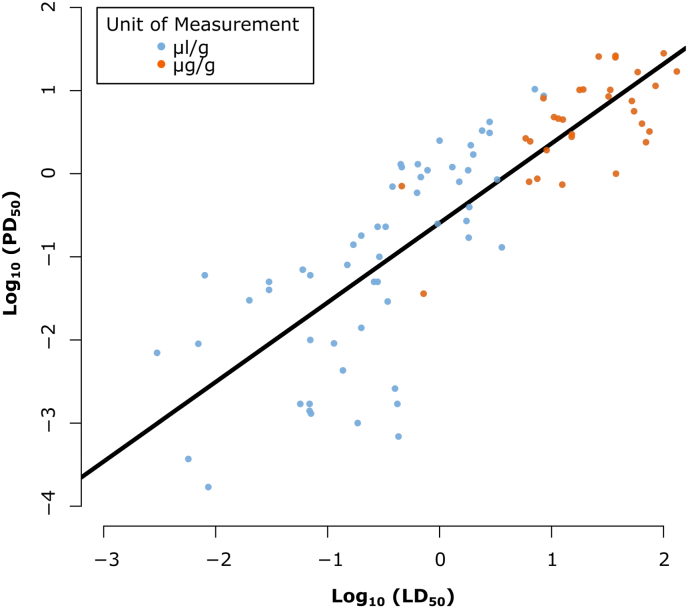
**Relationship between log_10_ of LD_50_ and log_10_ of PD_50_ for 89 measures of LD_50_ and 89 measures of PD_50_ across 55 species venoms spanning 26 different families**, performed on 12 different prey models, including eight insects, three arachnids and one crustacean model. The potency values were calculated in microliter of venom per gram of prey (μl/g in blue) or in microgram of venom per gram of prey (μg/g in orange) and included both data produced in the lab for 12 species and literature based data. PD_50_ values were calculated using various endpoints ranging from 30 min to 72 h but mostly with endpoints between 1 h and 4 h (S2). The fitted line highlights the significant positive, linear relationship between log_10_ LD_50_ and log_10_ PD_50_ (β (slope) = 0.96, lower 95% CI = 0.82, upper 95% CI = 1.1; S5: Table M1). (For interpretation of the references to colour in this figure legend, the reader is referred to the Web version of this article.)

We found support for a near isometric relationship between PD_50_ and LD_50_ measures of spider venom potency (β (slope) = 0.96, lower 95% CI = 0.82, upper 95% CI = 1.1; Fig. 1, S5: Table M1). Hence, when LD_50_ increases, PD_50_ increases approximately by the same amount. The model had an intercept of −0.6, which indicates that the venom quantities required to incapacitate prey are approximately fourfold lower than the quantities required for lethal effects. We observed a h^2^ value is 0.003, which indicates that there is a low phylogenetic signal.

We ran four supplementary models, three GLMs (S5: Table S1–S3) and one MCMCglmm (S5: Table S4). In GLM S1, we also found support for an isometric relationship between LD_50_ mg/kg and PD_50_ mg/kg (4 h) measures of spider venom potency (β = 1.01, SE = 0.24, p value < 0.05, n = 48 venom potency measures for 12 species; S5: Table S1). While the intercepts differed between prey models in GLM S1, these differences were not significant (β = −0.48, SE = 0.35, p value = 0.18; S5: Table S1). In GLM S2, the relationship between LD_50_ mg/kg and PD_50_ mg/kg was also near isometric when PD_50_ mg/kg was calculated using the 1-h endpoint (β = 0.95, SE = 0.23, p value = < 0.05, n = 46 venom potency measures for 11 species; S5: Table S2). In GLM S3, excluding *P. phalangioides* due to it requiring a different venom extraction method from the other 11 species in our lab based data did not change the observed isometric relationship between LD_50_ mg/kg and PD_50_ mg/kg (4 h) measures of spider venom potency (β = 1.00, SE = 0.25, p value = < 0.05, n = 46 venom potency measures for 11 species; S5: Table S3). Finally, for MCMCglmm S4 which only incorporated data collated from the literature, we also found support for a near isometric relationship between LD_50_ and PD_50_ measures of spider venom potency (S2) (β (slope) = 0.98, lower 95% CI = 0.79, upper 95% CI = 1.12; S5: Table S4).

## 4 Discussion

The results of both our main and supplementary analyses demonstrate that spider venom potency measures, PD_50_ and LD_50_, have a near isometric, positive relationship. This suggests that spider venom PD_50_ and LD_50_ values are generally close proxies of one another, with an increase in paralysis efficiency resulting in a similar increase in lethality. Furthermore, we did not find support for the sublinear scaling that would indicate lethality is decoupled from the paralysing ability of venoms in any of our analyses. Instead, our results suggests that either selection for lethality is comparable to the level of selection for paralysis efficiency, or that this correlation is a consequence of functionally coupled toxins, with selection for toxin paralysis efficiency positively impacting the lethality of the same toxins due to the overlapping nature of these functions.

While our analyses tested the potency of whole spider venoms, with the modus operandi of each species venom requiring further investigation, it is possible that only specific toxins are functionally coupled between paralysis capacity and lethality. For example, several toxins are known to induce a gradual secondary paralysis after the initial rapid actions of other toxins, which can effectively lead to lethality. For example, the venoms of Australian funnel-web spiders (Atracidae) contain certain peptidic blockers of insect voltage-gated calcium channels that can induce a gradual, flaccid paralysis within 20 - 30 min (Chong et al., 2007; King and Hardy, 2013). While such slow acting toxins may have been selected for their ability to prolong incapacitating effects in prey, they may also be tightly linked to later lethal effects, as their effects have been shown to be irreversible and typically target vital functions, which can ultimately lead to organ failure (King and Hardy, 2013; Langenegger et al., 2019; Bolon et al., 2023).

Alternatively, the linear isometric relationship found here could indicate strong selection for slower acting toxins with lethal effects. For example, prolonged paralytic effects that result in prey mortality may be selected for in species that engage in food storage behaviours (Griffiths et al., 2003; Dippenaar-Schoeman and Harris, 2005; Petráková et al., 2015), bite and run tactics (Pekár, 2004; Pekár et al., 2014) or extensive feeding sessions on relatively large prey, or vertebrate prey, which can last from several hours to several days (Nyffeler and Gibbons, 2022). However, the modus operandi for many spider venom toxins is still relatively unexplored, with such slower acting toxins only being observed in a few species (Adams, 2004; Herzig and Hodgson, 2008; Michálek et al., 2019). Furthermore, the strong correlation observed here between PD_50_ and LD_50_ may partly reflect the shared experimental system implemented to calculate the values rather than an intrinsic coupling between paralysis efficiency and lethality. Hence, whether the linear relationship found here is due to some mechanistic coupling, direct selection pressures or shared experimental conditionals or mathematical structure is unclear and will require more data on the specific actions of toxins across a broader range of species.

Whether it is due to strong coupling, selection for prolonged effects in venoms, or is the outcome of some unknown shared experimental or mathematical structures, the isometric relationship between LD_50_ and PD_50_ observed here indicates that both measures are good proxies for one another, at least in spiders. While PD_50_ is regarded as the more ecologically relevant measure, available datasets remain sparse in the literature, for all venomous taxa. However, LD_50_ measures for spider venoms are far more prevalent (Pekár et al., 2021) and these could be used as tentative proxies to address both species-specific and large-scale questions related to venom potency. Our analyses support the results of previous studies that used LD_50_ values to test hypotheses related to spider venom potency (Wigger et al., 2002; Herzig et al., 2004; Eggs et al., 2015; Clémençon et al., 2020) and the idea that both LD_50_ and PD_50_ values can be used to identify ecological patterns in spider venoms, such as prey-specific potency (Michálek et al. 2019, 2022; Valenzuela-Rojas et al., 2019; Lyons et al., 2025). However, whether our findings extend to other venomous groups is unclear. Specifically, future studies focusing on venomous groups that are unlikely to benefit from long-term or lethal effects are expected to offer further insight into whether lethality is generally functionally coupled with paralysis efficiency or is more directly selected for in predation-oriented venoms.

Overall, our results indicate that the spider venom potency measures PD_50_ and LD_50_, as well as the functions they measure, have a positive, linear isometric relationship, meaning that selection for increased paralysis efficiency results in a similar increase for venom lethality. This relationship suggests: 1) that spider venom toxins responsible for incapacitating prey (especially prolonged paralytic effects) may potentially be functionally coupled with lethality and 2) that PD_50_ and LD_50_ are good proxies for one another, meaning historically available LD_50_ values may be used to compare general spider venom potencies, provided they are based on the same prey model.

## CRediT authorship contribution statement

**Keith Lyons:** Writing – original draft, Visualization, Software, Methodology, Investigation, Funding acquisition, Formal analysis, Data curation, Conceptualization. **Dayle Leonard:** Writing – review & editing, Methodology, Investigation, Formal analysis, Data curation. **Leona McSharry:** Writing – review & editing, Investigation, Formal analysis, Data curation. **Michael Martindale:** Writing – review & editing, Investigation, Formal analysis, Data curation. **Brandon L. Collier:** Writing – review & editing, Investigation, Formal analysis, Data curation. **Aiste Vitkauskaite:** Writing – review & editing, Investigation, Formal analysis, Data curation. **John P. Dunbar:** Writing – review & editing, Investigation, Formal analysis, Data curation. **Michel M. Dugon:** Writing – review & editing, Visualization, Supervision, Resources, Methodology, Investigation, Funding acquisition, Formal analysis, Data curation, Conceptualization. **Kevin Healy:** Writing – review & editing, Visualization, Validation, Supervision, Software, Resources, Methodology, Investigation, Funding acquisition, Formal analysis, Data curation, Conceptualization.

## Funding

K.L. was funded by an 10.13039/501100002081Irish Research Council Postgraduate Scholarship (GOIPG/2020/961). Part of this research was also funded by the 10.13039/501100001634University of Galway, Thomas Crawford Hayes Research Award, awarded to K.L. in 2020.

## Declaration of competing interest

The authors declare that they have no known competing financial interests or personal relationships that could have appeared to influence the work reported in this paper.

## Data Availability

All data and code are included in the supplementary files S1 – S5 that have been submitted alongside this manuscript.
