# Corrigendum to “Paralysis efficiency (PD_50_) scales linearly with lethality (LD_50_) in spider venoms” [Toxicon: X. Volume 30 (2026). 100256]

**DOI:** 10.1016/j.toxcx.2026.100257

**Published:** 2026-05-13

**Authors:** Keith Lyons, Dayle Leonard, Leona McSharry, Michael Martindale, Brandon L. Collier, Aiste Vitkauskaite, John P. Dunbar, Michel M. Dugon, Kevin Healy

**Affiliations:** a Macroecology Lab, School of Natural Sciences, Ryan Institute, University of Galway, Galway, H91 TK33, Ireland; b Venom Systems & Proteomics Lab, School of Natural Sciences, Ryan Institute, University of Galway, Galway, H91 TK33, Ireland; c Institute for Insect Biotechnology, Faculty for Agricultural Sciences, Nutritional Sciences, and Environmental Management, Justus Liebig University Giessen, Heinrich-Buff-Ring 58, Giessen, 35392, Germany; d Midlands Bug and Reptile Zoo, Longford, Ireland

The authors regret.

Two points:

1) In Table 2, in the reference column, “Nentwig et al., 1992” and “Friedel and Nentwig, 1989” currently do not have a hyperlink connecting them to their associated full reference as the others do. This should be changed so that they do.
2) “Caitlin” in the Acknowledgements section should instead be “Kathleen”.

No changes are required outside of the two points above. The authors would like to apologise for any inconvenience caused.

