# Supplementary material for: Paralysis efficiency (PD50) scales linearly with lethality (LD50) in spider venoms
Source: Toxicon X. 2026 Apr 17;30:100256. doi: 10.1016/j.toxcx.2026.100256 (PMC13138031; doi:10.1016/j.toxcx.2026.100256)
Supplement: Multimedia component 5 [file mmc5.docx]

**Paralysis Efficiency (PD_50_) Scales Linearly With Lethality (LD_50_) in Spider Venoms**

**Keith Lyons^abc^*†, Dayle Leonard^b^†, Leona McSharry^b^, Michael Martindale^b^, Brandon L. Collier^b^, Aiste Vitkauskaite^b^, John P. Dunbar^d^, Michel M. Dugon^b^† & Kevin Healy^a^†**

*****Corresponding Author

† These authors contributed equally to this work.

^a^Macroecology lab, School of Natural Sciences, Ryan Institute, University of Galway, H91 TK33 Galway, Ireland.

^b^Venom Systems & Proteomics Lab, School of Natural Sciences, Ryan Institute, University of Galway, H91 TK33 Galway, Ireland.

^c^ Institute for Insect Biotechnology, Faculty for Agricultural Sciences, Nutritional Sciences, and Environmental Management, Justus Liebig University Giessen, Heinrich-Buff-Ring 58, 35392 Giessen, Germany.

^d^Midlands Bug and Reptile Zoo, Longford, Ireland.

**ORCID ID**: **K.L.** 0000-0002-3572-0847, **D.L.** 0000-0002-2943-748X, **L.McS.** 0000-0002-8823-1828, **M.M.** 0009-0005-4235-9059, **B.L.C.** 0000-0002-0709-9678, **A.V.** 0000-0001-9148-0916, **J.P.D.** 0000-0002-6645-0472, **M.M.D.** 0000-0002-8567-819X, **K.H.** 0000-0002-3548-6253.

**Supplementary S5: Supplementary Tables and Model Outputs**


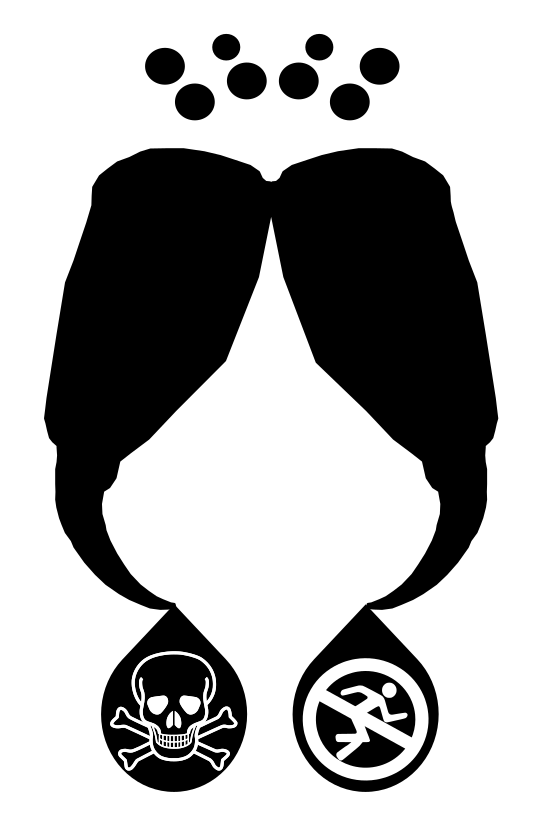


| **Table M1: Output for the Main MCMCglmm testing the relationship between log_10_ of LD_50_ (μl/g) and log_10_ of PD_50_ (μl/g)** for 89 measures of LD_50_ and 89 measures of PD_50_ across 55 species, spanning 26 families, in 12 prey models (eight insects, three arachnids & one crustacean). The modes (β) and standard error range are provided for both fixed and random terms, with log_10_ PD_50_ as the response variable and log_10_ LD_50_ as the fixed term. The random terms associated with phylogenetic relatedness (phylogeny (h^2^)), intraspecific variation (species) and residual variation (residual) are also presented. Significant values, which are highlighted in **bold**, are those with 95% of the posterior estimate above or below zero. | | | |
| --- | --- | --- | --- |
|  | **β** | **95% Lower CI** | **95% Upper CI** |
| **Fixed Terms** |  |  |  |
| Intercept _Log10_ PD_50 (μl/g)_ | **-0.59** | **-0.86** | **-0.35** |
| Log_10_ LD_50_ (μl/g) | **0.96** | **0.82** | **1.1** |
|  |  |  |  |
| **Random Terms** |  |  |  |
| Phylogeny (h^2^) | 0.003 | 0.0003 | 0.24 |
| Species | 0.003 | 0.0003 | 0.27 |
| Residuals | 0.99 | 0.62 | 1.00 |

| **Table S1: Output for Supplementary GLM 1 (S1) testing the relationship between log_10_ of** LD_50_ **(mg/kg) and log_10_ of** PD_50_ **(mg/kg)** for 24 measures of LD_50_ and 24 measures of PD_50_ (4 hr endpoint) across 12 species in two prey models, *Acheta domesticus* and *Porcellio scaber*. The modes (β) and standard error range are provided for both fixed and random terms, with log_10_ PD_50_ as the response variable and log_10_ LD_50_ as the fixed term. Results are deemed significant when p-value < 0.05 and are highlighted in **bold**. | | | | |
| --- | --- | --- | --- | --- |
|  | **β** | **Standard error** | **t value** | **P value** |
| **Fixed Terms** |  |  |  |  |
| Intercept _log10_ PD_50 (mg/kg)_ | **-0.82** | **0.28** | **-2.93** | **0.01** |
| Log_10_ LD_50_ (mg/kg) | **1.01** | **0.24** | **4.21** | **0.0004** |
| Prey model *_Porcellio scaber_* | 1.02 | 0.5 | 2.04 | 0.055 |
| Log_10_ LD_50_ (mg/kg): Prey model *_Porcellio scaber_* | -0.48 | 0.35 | -1.37 | 0.18 |


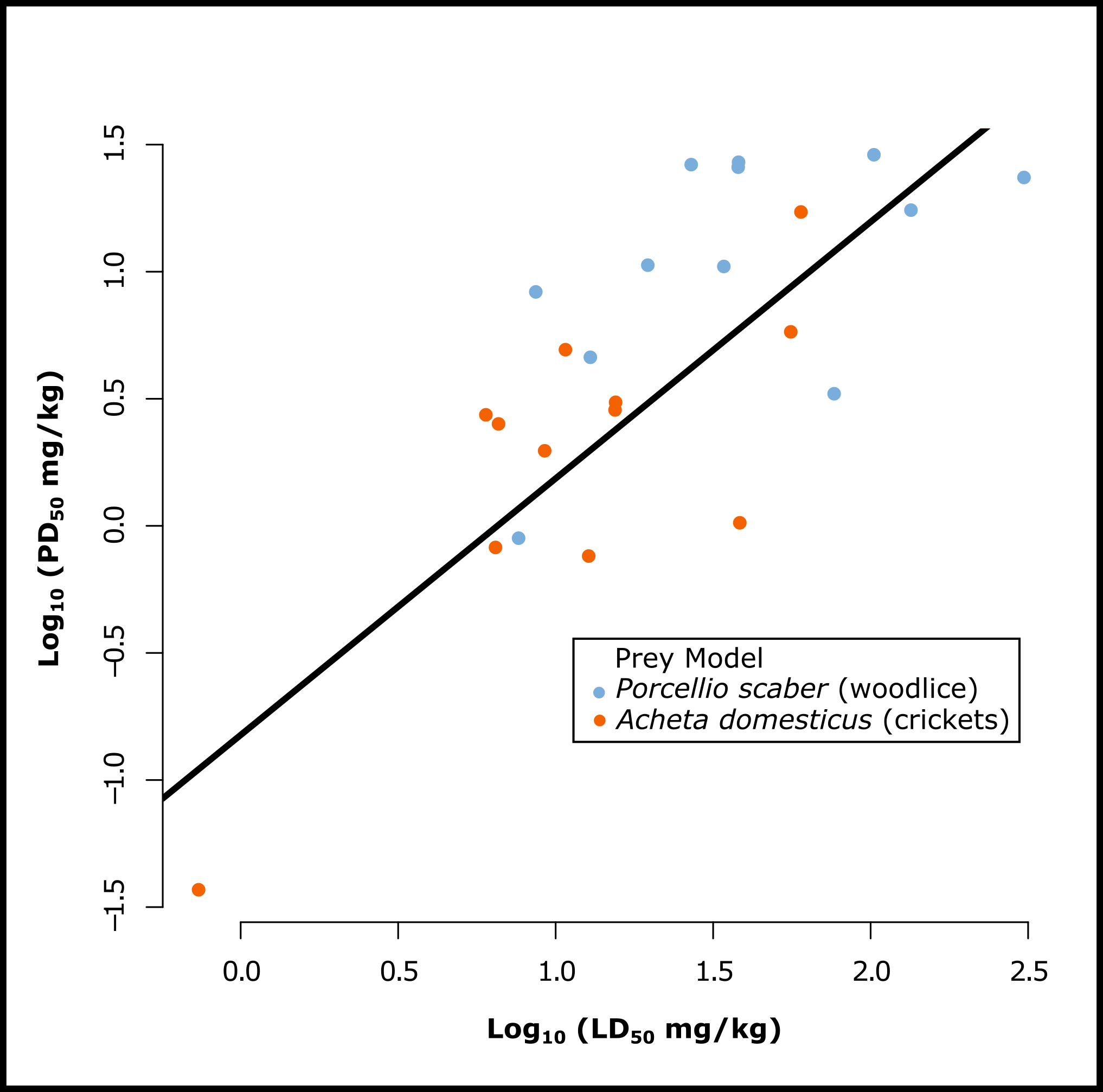


**Figure S1: Relationship between log_10_ of LD_50_ (mg/kg) and log_10_ of** PD_50_ **(mg/kg)** for 24 measures of LD_50_ and 24 measures of PD_50_ (4 hr endpoint) across 12 species venoms, tested in two prey models, *A. domesticus* (orange) and *P. scaber* (blue). The fitted line highlights the significant positive, isometric relationship between log_10_ LD_50_ (mg/kg) and log_10_ PD_50_ (mg/kg) (4 hr) (β = 1.01, SE = 0.24, p value = 0.0004; S5: Table S1).

| **Table S2: Output for Supplementary GLM 2 (S2) testing the relationship between log_10_ of LD_50_ (mg/kg) and log_10_ of PD_50_ (mg/kg)** for 23 measures of LD_50_ and 23 measures of PD_50_ (1 hr endpoint) across 11 species (*Cupiennius coccineus* excluded), in two prey models, *Acheta domesticus* and *Porcellio scaber*. The modes (β) and standard error range are provided for both fixed and random terms, with log_10_ PD_50_ as the response variable and log_10_ LD_50_ as the fixed term. Results are deemed significant when p ≤ 0.05 and are highlighted in **bold**. | | | | |
| --- | --- | --- | --- | --- |
|  | **β** | **Standard error** | **t value** | **P value** |
| **Fixed Terms** |  |  |  |  |
| Intercept _log10_ PD_50 (mg/kg)_ | **-0.73** | **0.28** | **-2.65** | **0.02** |
| Log_10_ LD_50_ (mg/kg) | **0.95** | **0.23** | **4.06** | **0.0007** |
| Prey model *_Porcellio scaber_* | **1.27** | **0.49** | **2.57** | **0.02** |
| Log_10_ LD_50_ (mg/kg): Prey model  *_Porcellio scaber_* | -0.57 | 0.34 | -1.66 | 0.11 |


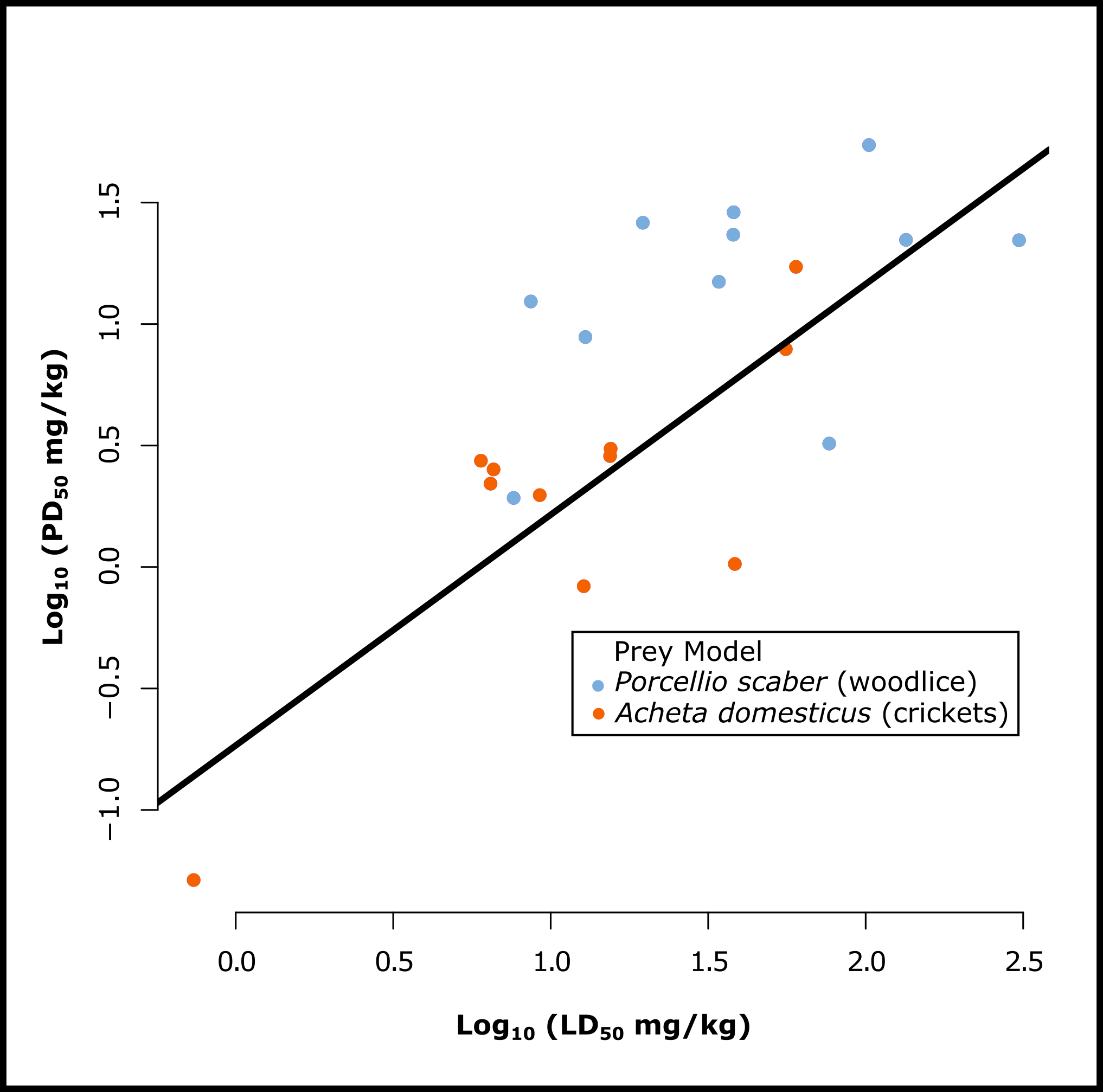


**Figure S2:** Relationship between log_10_ of LD_50_ (mg/kg) and log_10_ of PD_50_ (mg/kg) for 23 measures of LD_50_ and 23 measures of PD_50_ (1 hr endpoint) across 11 species in two prey models, *A. domesticus* (orange) and *P. scaber* (blue). The fitted orange line highlights the significant positive, isometric relationship between log_10_ LD_50_ (mg/kg) and log_10_ PD_50_ (mg/kg)(1 hr) in *A. domesticus* (β = 0.95, SE = 0.23, p value = 0.0007: Table S1). See Table S7 of this document for a table detailing the PD_50_ and LD_50_ values calculated using the 1 hr endpoint.

| **Table S3: Output for Supplementary GLM 3 (S3) testing the relationship between log_10_ of LD_50_ (mg/kg) and log_10_ of PD_50_ (mg/kg)** for 23 measures of LD_50_ and 23 measures of PD_50_ (4 hr endpoint) across 11 species (*Pholcus phalangioides* excluded as their venom was extracted via venom gland extraction), in two prey models, *Acheta domesticus* and *Porcellio scaber*. The modes (β) and standard error range are provided for both fixed and random terms, with log_10_ PD_50_ as the response variable and log_10_ LD_50_ as the fixed term. Results are deemed significant when p ≤ 0.05 and are highlighted in **bold**. | | | | |
| --- | --- | --- | --- | --- |
|  | **β** | **Standard error** | **t value** | **P value** |
| **Fixed Terms** |  |  |  |  |
| Intercept _log10_ PD_50 (mg/kg)_ | **-0.81** | **0.31** | **-2.64** | **0.02** |
| Log_10_ LD_50_ (mg/kg) | **1.00** | **0.25** | **3.93** | **0.001** |
| Prey model *_Porcellio scaber_* | 1.06 | 0.56 | 1.90 | 0.07 |
| Log_10_ LD_50_ (mg/kg): Prey model  *_Porcellio scaber_* | -0.55 | 0.34 | -1.63 | 0.12 |


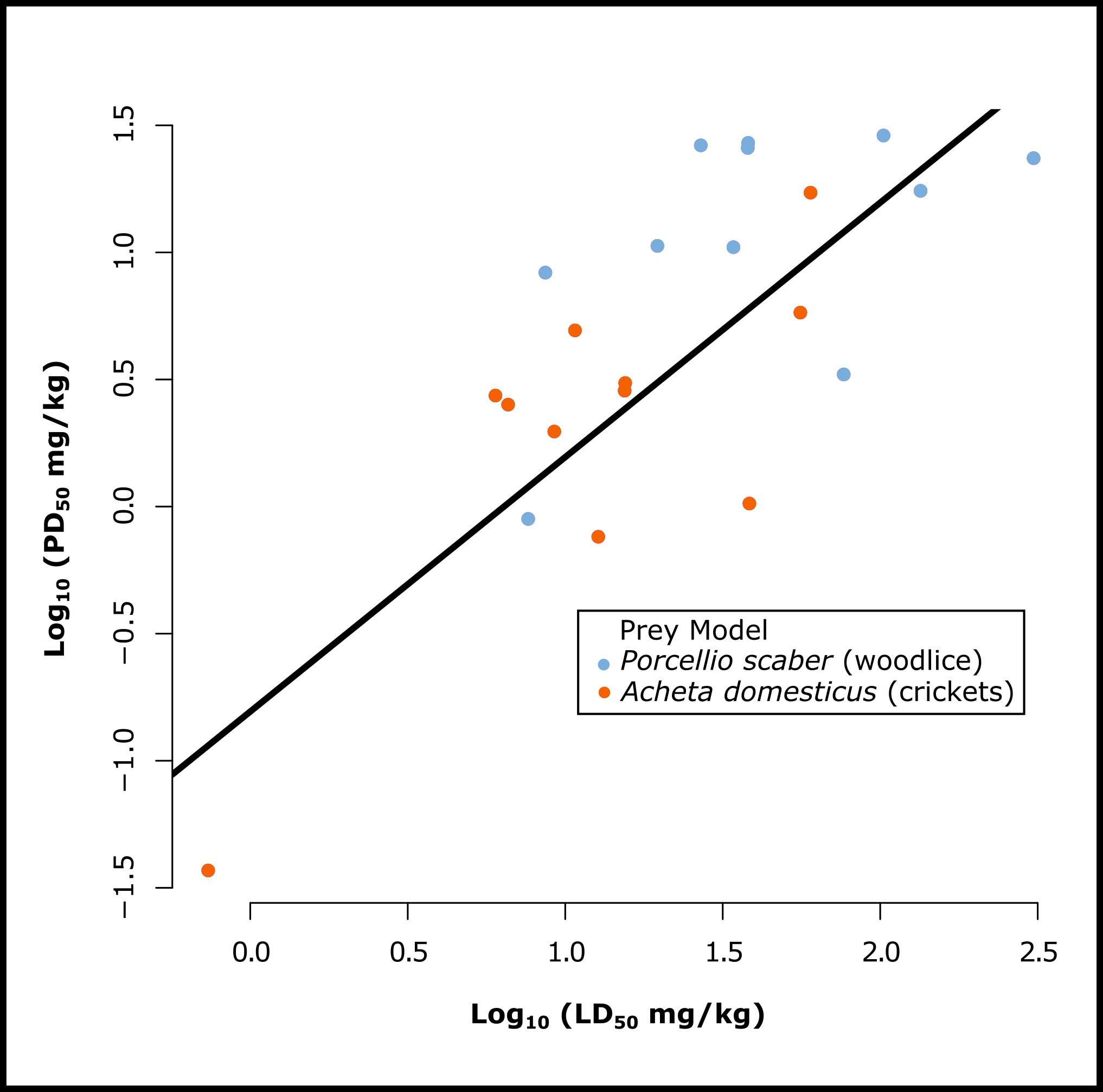


**Figure S3: Relationship between log_10_ of** LD_50_ **(mg/kg) and log_10_ of** PD_50_ **(mg/kg)** for 23 measures of LD_50_ and 23 measures of PD_50_ (4 hr endpoint) across 11 species (*P. phalangioides* excluded) in two prey models, *A. domesticus* (orange) and *P. scaber* (blue). The fitted orange line highlights the significant positive, isometric relationship between log_10_ LD_50_ (mg/kg) and log_10_ PD_50_ (mg/kg)(1 hr) in *A. domesticus* (β = 1.0, SE = 0.25, p value = 0.001: Table S3).

| **Table S4: Output for the Supplementary MCMCglmm (S4) testing the relationship between log_10_ of LD_50_ (μl/g) and log_10_ of PD_50_ (μl/g)** for 65 measures of LD_50_ and 65 measures of PD_50_ across 46 species in 11 prey models (eight insects, three arachnids). The modes (β) and standard error range are provided for both fixed and random terms, with log_10_ PD_50_ as the response variable and log_10_ LD_50_ as the fixed term. The random terms associated with phylogenetic relatedness (phylogeny (h^2^)), intraspecific variation (species) and residual variation (residual) are also presented. Significant values, which are highlighted in **bold**, are those with 95% of the posterior estimate above or below zero. | | | |
| --- | --- | --- | --- |
|  | **β** | **95% Lower CI** | **95% Upper CI** |
| **Fixed Terms** |  |  |  |
| Intercept _Log10_ PD_50 (μl/g)_ | **-0.58** | **-0.9** | **-0.27** |
| Log_10_ LD_50_ (μl/g) | **0.98** | **0.79** | **1.12** |
|  |  |  |  |
| **Random Terms** |  |  |  |
| Phylogeny (h^2^) | 0.002 | 0.0002 | 0.27 |
| Species | 0.002 | 0.0002 | 0.24 |
| Residuals | 0.99 | 0.63 | 1.00 |


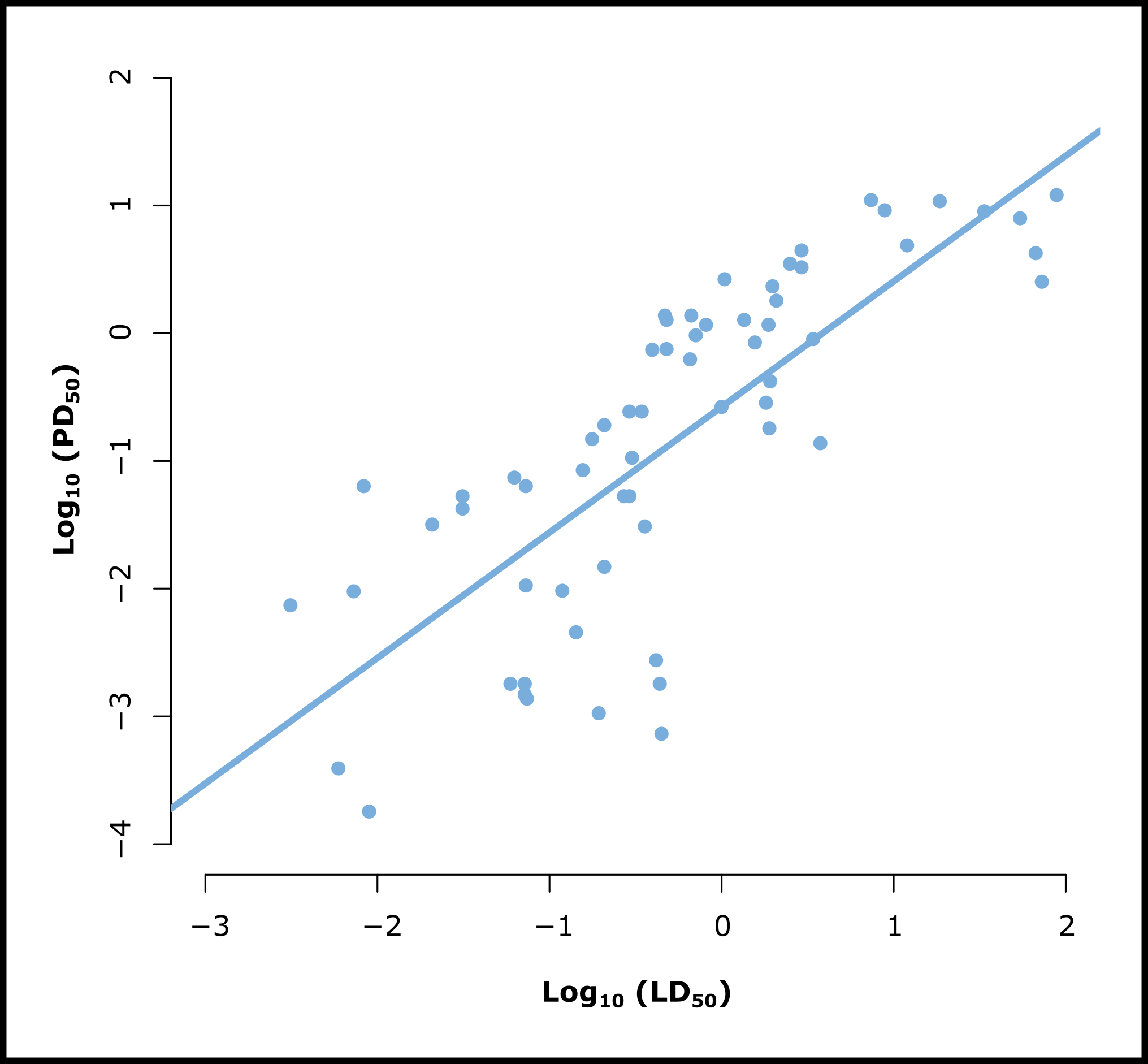


**Figure S4: Relationship between log_10_ of LD_50_ (μl/g) and log_10_ of** PD_50_ **(μl/g)** for 65 measures of LD_50_ and 65 measures of PD_50_ across 46 species venoms spanning 23 different families, performed on 11 different prey models, including eight insect models and three arachnid model (S2). The fitted line highlights the significant positive, linear relationship between log_10_ LD_50_ (μl/g) and log_10_ PD_50_ (μl/g) (β (slope) = 0.98, lower 95% CI = 0.79, upper 95% CI = 1.12; Table S4).

| **Table S5: Body length and venom yield data for the 12 spider species tested in the lab.** The data presented includes the number of specimens used for venom extraction, full spider body length (mm), the total liquid venom yield (μl) extracted, the total venom yield extracted expressed in milligram of lyophilised protein content per microliter of venom (mg/μl) and the venom yield expressed in milligram of dried venom extracted per specimen (mg/spider). | | | | | |
| --- | --- | --- | --- | --- | --- |
| Spider Species | Number of Specimens Extracted | Mean Full Body Length (mm) | Total Venom Yield (μl) | Total Venom Yield (mg/μl)  (100% Concentration) | Venom Yield (mg/spider) |
| *Amaurobius similis* | 117 | 9.5 | 13.9 | 0.07908 | 0.0094 |
| *Cupiennius coccineus* | 5 (2 twice) | 16.3 | 4.7 | 0.05920 | 0.04 |
| *Cupiennius salei* | 6 (4 twice) | 15.3 | 8.0 | 0.04720 | 0.038 |
| *Eratigena atrica* | 99 | 12.0 | 42.3 | 0.15120 | 0.065 |
| *Heteropoda venatoria* | 10 (4 twice) | 18.3 | 44.5 | 0.10325 | 0.33 |
| *Larinioides sclopetarius* | 81 | 7.2 | 7.2 | 0.23152 | 0.0205 |
| *Meta menardi* | 63 | 11.6 | 15.7 | 0.07619 | 0.019 |
| *Monocentropus balfouri* | 4 | 46.4 | 19.9 | 0.11055 | 0.55 |
| *Pholcus phalangioides* | 140 | 6.9 | 7.9 | 0.08228 | 0.0046 |
| *Phormictopus cancerides* | 1 | 48.9 | 9.9 | 0.18429 | 1.83 |
| *Piloctenus haematostoma* | 4 | 26.5 | 30.3 | 0.00990 | 0.075 |
| *Steatoda nobilis* | 117 | 9.8 | 14.2 | 0.09880 | 0.012 |

| **Table S6: Shows the venom concentrations that were tested for 12 spider species venoms during the bioassay experiments in both prey models**, House cricket *Acheta domesticus* and Common Rough woodlouse *Porcellio scaber* (n = 20 per cohort). Also presented are the number of spiders extracted to obtain each species venom sample, the total crude venom extracted (μl) and the 100% venom yield concentration obtained, expressed in milligram of lyophilised (dried) venom per microliter of venom (mg/μl). | | | | | |
| --- | --- | --- | --- | --- | --- |
| Spider Species | Number of Specimens Extracted | Total Venom Yield (μl) | Total Venom Yield (mg/μl)  (100% Concentration) | Venom Concentration Injected (mg/μl) | Venom Concentration Injected  (% Concentration) |
| *Amaurobius similis* | 117 | 13.91 | 0.07908 | 0.003164 | 4 |
| *Amaurobius similis* | 117 | 13.91 | 0.07908 | 0.000791 | 1 |
| *Amaurobius similis* | 117 | 13.91 | 0.07908 | 0.000158 | 0.2 |
| *Amaurobius similis* | 117 | 13.91 | 0.07908 | 0.000079 | 0.1 |
| *Amaurobius similis* | 117 | 13.91 | 0.07908 | 0.000039 | 0.05 |
| *Amaurobius similis* | 117 | 13.91 | 0.07908 | 0.000008 | 0.01 |
| *Cupiennius coccineus* | 5 (2 twice) | 4.73 | 0.05920 | 0.001184 | 2 |
| *Cupiennius coccineus* | 5 (2 twice) | 4.73 | 0.05920 | 0.000118 | 0.2 |
| *Cupiennius coccineus* | 5 (2 twice) | 4.73 | 0.05920 | 0.000012 | 0.02 |
| *Cupiennius salei* | 6 (4 twice) | 8.05 | 0.04720 | 0.001416 | 3 |
| *Cupiennius salei* | 6 (4 twice) | 8.05 | 0.04720 | 0.000236 | 0.5 |
| *Cupiennius salei* | 6 (4 twice) | 8.05 | 0.04720 | 0.000047 | 0.1 |
| *Cupiennius salei* | 6 (4 twice) | 8.05 | 0.04720 | 0.000005 | 0.01 |
| *Eratigena atrica* | 99 | 42.33 | 0.15120 | 0.030239 | 20 |
| *Eratigena atrica* | 99 | 42.33 | 0.15120 | 0.01512 | 10 |
| *Eratigena atrica* | 99 | 42.33 | 0.15120 | 0.006048 | 4 |
| *Eratigena atrica* | 99 | 42.33 | 0.15120 | 0.003024 | 2 |
| *Eratigena atrica* | 99 | 42.33 | 0.15120 | 0.001512 | 1 |
| *Eratigena atrica* | 99 | 42.33 | 0.15120 | 0.000756 | 0.5 |
| *Eratigena atrica* | 99 | 42.33 | 0.15120 | 0.000151 | 0.1 |
| *Heteropoda venatoria* | 10 (4 twice) | 44.55 | 0.10325 | 0.008264 | 8 |
| *Heteropoda venatoria* | 10 (4 twice) | 44.55 | 0.10325 | 0.004132 | 4 |
| *Heteropoda venatoria* | 10 (4 twice) | 44.55 | 0.10325 | 0.001033 | 1 |
| *Heteropoda venatoria* | 10 (4 twice) | 44.55 | 0.10325 | 0.0005165 | 0.5 |
| *Heteropoda venatoria* | 10 (4 twice) | 44.55 | 0.10325 | 0.0001033 | 0.1 |
| *Heteropoda venatoria* | 10 (4 twice) | 44.55 | 0.10325 | 0.00001033 | 0.01 |
| *Larinioides sclopetarius* | 81 | 7.17 | 0.23152 | 0.002315 | 1 |
| *Larinioides sclopetarius* | 81 | 7.17 | 0.23152 | 0.0011575 | 0.5 |
| *Larinioides sclopetarius* | 81 | 7.17 | 0.23152 | 0.0002315 | 0.1 |
| *Meta menardi* | 63 | 15.75 | 0.07619 | 0.003048 | 4 |
| *Meta menardi* | 63 | 15.75 | 0.07619 | 0.000762 | 1 |
| *Meta menardi* | 63 | 15.75 | 0.07619 | 0.000571 | 0.75 |
| *Meta menardi* | 63 | 15.75 | 0.07619 | 0.000381 | 0.5 |
| *Meta menardi* | 63 | 15.75 | 0.07619 | 0.000076 | 0.1 |
| *Meta menardi* | 63 | 15.75 | 0.07619 | 0.000008 | 0.01 |
| *Monocentropus balfouri* | 4 | 19.9 | 0.11055 | 0.006633 | 6 |
| *Monocentropus balfouri* | 4 | 19.9 | 0.11055 | 0.002211 | 2 |
| *Monocentropus balfouri* | 4 | 19.9 | 0.11055 | 0.001105 | 1 |
| *Monocentropus balfouri* | 4 | 19.9 | 0.11055 | 0.000553 | 0.5 |
| *Monocentropus balfouri* | 4 | 19.9 | 0.11055 | 0.000111 | 0.1 |
| *Monocentropus balfouri* | 4 | 19.9 | 0.11055 | 0.000011 | 0.01 |
| *Pholcus phalangioides* | 140 | 7.9 | 0.08228 | 0.001646 | 2 |
| *Pholcus phalangioides* | 140 | 7.9 | 0.08228 | 0.000823 | 1 |
| *Pholcus phalangioides* | 140 | 7.9 | 0.08228 | 0.000411 | 0.5 |
| *Pholcus phalangioides* | 140 | 7.9 | 0.08228 | 0.000041 | 0.05 |
| *Phormictopus cancerides* | 1 | 9.93 | 0.18429 | 0.005529 | 3 |
| *Phormictopus cancerides* | 1 | 9.93 | 0.18429 | 0.0027645 | 1.5 |
| *Phormictopus cancerides* | 1 | 9.93 | 0.18429 | 0.001843 | 1 |
| *Phormictopus cancerides* | 1 | 9.93 | 0.18429 | 0.0009215 | 0.5 |
| *Phormictopus cancerides* | 1 | 9.93 | 0.18429 | 0.0001843 | 0.1 |
| *Piloctenus haematostoma* | 4 | 30.29 | 0.00990 | 0.00099 | 10 |
| *Piloctenus haematostoma* | 4 | 30.29 | 0.00990 | 0.000198 | 2 |
| *Piloctenus haematostoma* | 4 | 30.29 | 0.00990 | 0.000099 | 1 |
| *Piloctenus haematostoma* | 4 | 30.29 | 0.00990 | 0.0000099 | 0.1 |
| *Steatoda nobilis* | 117 | 14.17 | 0.09880 | 0.002964 | 3 |
| *Steatoda nobilis* | 117 | 14.17 | 0.09880 | 0.000988 | 1 |
| *Steatoda nobilis* | 117 | 14.17 | 0.09880 | 0.000494 | 0.5 |
| *Steatoda nobilis* | 117 | 14.17 | 0.09880 | 0.000099 | 0.1 |
| *Steatoda nobilis* | 117 | 14.17 | 0.09880 | 0.000049 | 0.05 |
| *Steatoda nobilis* | 117 | 14.17 | 0.09880 | 0.000025 | 0.025 |
| *Steatoda nobilis* | 117 | 14.17 | 0.09880 | 0.000012 | 0.0125 |
| *Steatoda nobilis* | 117 | 14.17 | 0.09880 | 0.000006 | 0.00625 |

| **Table S7: Calculated potency measures for 12 spider species venoms** tested on house cricket *A. domesticus* and woodlouse *P. scaber*. Presented are the **PD_50_ (mg/kg) (1 hr endpoint)** and **LD_50_ (mg/kg) (24 hr endpoint)** values for the venom of each species, in both prey models, with standard errors, calculated using the dried venom protein mass (mg) and median prey model mass for crickets (0.0003 kg) or woodlice (0.00009 kg). Note *C. coccineus* venom failed to achieve a PD_50_ in woodlice using the 1 hr endpoint. *A. domesticus* data for *Eratigena atrica* was produced as part of Lyons et al. (2023). PD_50_ (1 hr) values that are different to their corresponding PD_50_ (4 hr) values are shown in **bold**. | | | | | | | | |
| --- | --- | --- | --- | --- | --- | --- | --- | --- |
|  | *Acheta domesticus* (cricket) | | | | *Porcellio scaber* (woodlouse) | | | |
| Spider Species | PD_50_ mg/kg | Standard Error | LD_50_ mg/kg | Standard Error | PD_50_ mg/kg | Standard Error | LD_50_ mg/kg | Standard Error |
| *Amaurobius similis* | 1.9 | 0.61 | 9.0 | 2.35 | **14.5** | **6.99** | 33.5 | 17.67 |
| *Cupiennius coccineus* | 4.8 | 1.24 | 10.5 | 6.71 | **na** | **na** | 26.4 | 10.86 |
| *Cupiennius salei* | 2.4 | 0.66 | 6.4 | 1.31 | **22.7** | **8.26** | 37.2 | 19.93 |
| *Eratigena atrica* | **7.7** | **2.53** | 54.6 | 10.66 | **21.5** | **25.87** | 300.5 | 50.23 |
| *Heteropoda venatoria* | 1.0 | 0.14 | 37.6 | 6.68 | **21.6** | **8.32** | 131.5 | 57.16 |
| *Larinioides sclopetarius* | 2.8 | 0.46 | 15.1 | 6.87 | **28.0** | **3.5** | 37.3 | 15.18 |
| *Meta menardi* | **0.8** | **0.20** | 12.5 | 2.74 | **3.1** | **3.46** | 75.0 | 58.5 |
| *Monocentropus balfouri* | 3.0 | 0.92 | 15.2 | 1.66 | **12.0** | **5.59** | 8.5 | 6.51 |
| *Pholcus phalangioides* | **2.1** | **0.44** | 6.3 | 2.25 | **8.6** | **1.28** | 12.6 | 1.56 |
| *Phormictopus cancerides* | 16.7 | 3.56 | 58.8 | 53.94 | **53.0** | **8.39** | 100.3 | 98.5 |
| *Piloctenus haematostoma* | 2.7 | 0.57 | 5.9 | 1.88 | **25.4** | **18.82** | 19.2 | 9.47 |
| *Steatoda nobilis* | **0.05** | **0.028** | 0.7 | 0.20 | **1.9** | **1.39** | 7.5 | 6.7 |

**Figure S5:** Phylogenetic tree produced using Supplementary S4 R studio code for 55 spider species in the S2 dataset and a base phylogenetic tree from ‘Wolff et al. (2022), *Systematic Biology’* (See main manuscript for full citation).


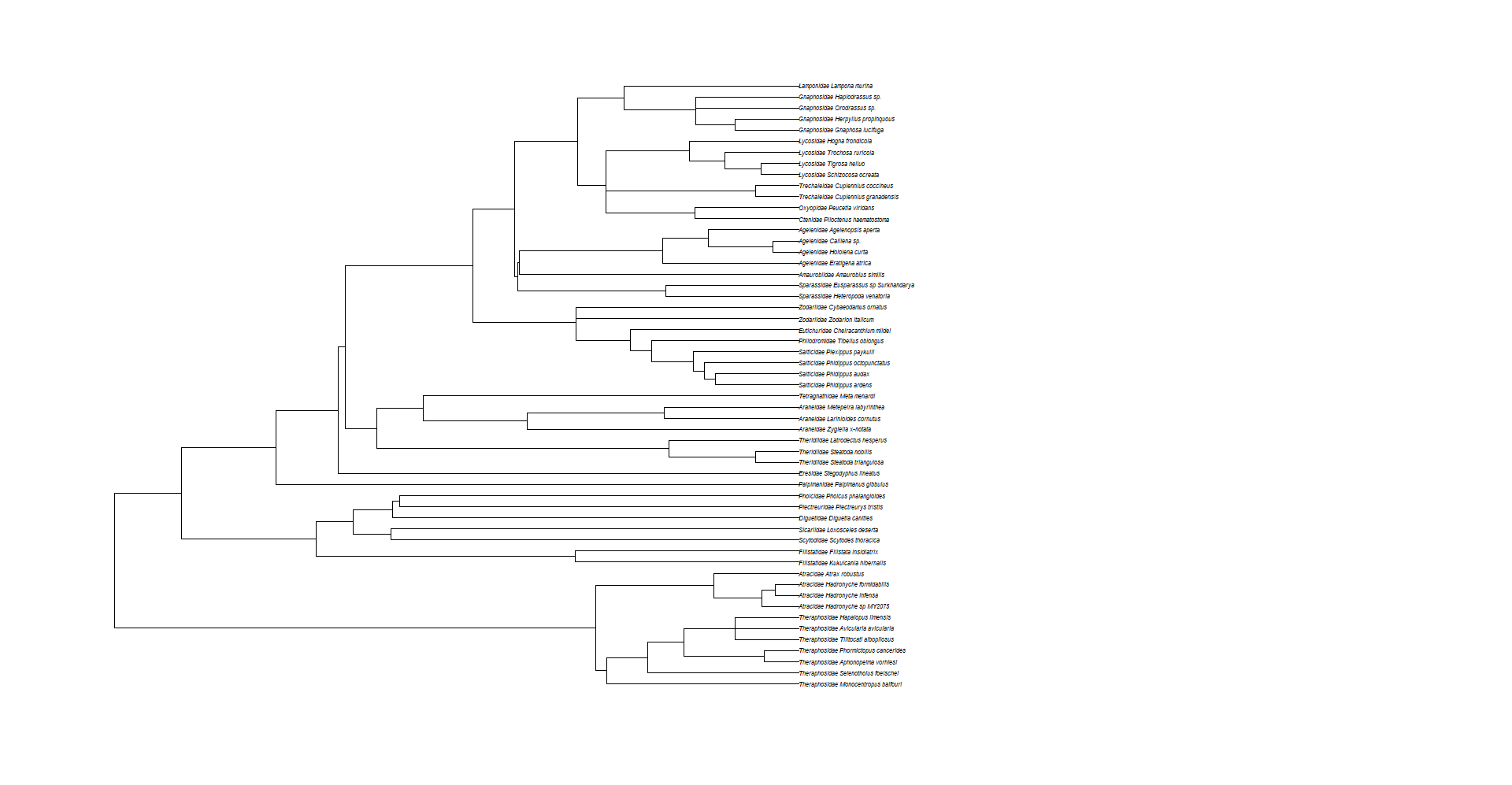


| **Table S8: List of species from Wolff et al. 2022 tree that were used as substitutions for species in our dataset, when species in our dataset were not present in the Wolff et al. 2022 tree.** There were 20 substitutions in total. The rationale for each choice is also provided. | | |
| --- | --- | --- |
|  | The Substitution from Wolff e al. 2022 tree | Rationale (any citations can be found in full in the main manuscript) |
| Species in our dataset being subbed |  |  |
| *Aphonopelma chalcodes* | *Aphonopelma vorhiesi* | Same Genus |
| *Cupiennius salei* | *Cupiennius granadensis* | Same Genus |
| *Cybaeodamus taim* | *Cybaeodamus ornatus* | Same Genus |
| *Diguetia albolineata* | *Diguetia albolineata* | Same Genus |
| *Filistata sp.* | *Filistata insidiatrix* | Same Genus |
| *Gnaphosa sp.* | *Gnaphosa lucifuga* | Same Genus |
| *Hadronyche cerberea* | *Hadronyche Sp MY2075* | Same Genus |
| *Hogna carolinensis* | *Hogna frondicola* | Same Genus |
| *Kukulkania sp.* | *Kukulcania hibernalis* | Same Genus |
| *Lampona sp.* | *Lampona murina* | Same Genus |
| *Larinioides sclopetarius* | *Larinioides cornutus* | Same Genus |
| *Metepeira_sp.* | *Metepeira labyrinthea* | Same Genus |
| *Olios sp.* | *Eusparassus sp Surkhandarya* | Same Family + not may representatives of this family in our dataset |
| *Palpimanus sp.* | *Palpimanus gibbulus* | Same Genus |
| *Phidippus johnsoni* | *Plexippus paykulli* | Guerrero-Fuentes et al 2024; Edwards 2004; Maddison 2003. |
| *Philodromid sp.* | *Tibellus oblongus* | Guerrero-Fuentes et al 2024; Azevedo et al 2018; Edwards 2004; Maddison 2003. |
| *Schizocosa mccooki* | *Schizocosa ocreata* | Same Genus |
| *Scytodes sp.* | *Scytodes thoracica* | Same Genus |
| *Steatoda grossa* | *Steatoda triangulosa* | Same Genus |
| *Trochosa sp.* | *Trochosa ruricola* | Same Genus |
